# The Impact of Pupil Constriction on the Relationship Between Melanopic EDI and Melatonin Suppression in Young Adult Males

**DOI:** 10.1177/07487304241226466

**Published:** 2024-02-13

**Authors:** Isabel Schöllhorn, Oliver Stefani, Robert J. Lucas, Manuel Spitschan, Christian Epple, Christian Cajochen

**Affiliations:** * Centre for Chronobiology, Psychiatric Hospital, University of Basel, Basel, Switzerland; † Research Platform Molecular and Cognitive Neurosciences (MCN), University of Basel, Basel, Switzerland; ‡ Lucerne University of Applied Sciences and Arts, Lucerne, Switzerland; § Centre for Biological Timing, School of Biology, Faculty of Biology, Medicine and Health, The University of Manchester, Manchester, UK; || Translational Sensory & Circadian Neuroscience, Max Planck Institute for Biological Cybernetics, Tübingen, Germany; ¶ Chronobiology & Health, Technical University of Munich, Munich, Germany; ** TUM Institute for Advanced Study (TUM-IAS), Technical University of Munich, Munich, Germany

**Keywords:** pupil, melatonin, melanopsin, silent substitution, non-visual effects of light

## Abstract

The pupil modulates the amount of light that reaches the retina. Not only luminance but also the spectral distribution defines the pupil size. Previous research has identified steady-state pupil size and melatonin attenuation to be predominantly driven by melanopsin, which is expressed by a unique subgroup of intrinsically photosensitive retinal ganglion cells (ipRGCs) that are sensitive to short-wavelength light (~480 nm). Here, we aimed to selectively target the melanopsin system during the evening, while measuring steady-state pupil size and melatonin concentrations under commonly experienced evening light levels (<90 lx). Therefore, we used a five-primary display prototype to generate light conditions that were matched in terms of L-, M-, and S-cone-opic irradiances, but with high and low melanopic irradiances (~3-fold difference). Seventy-two healthy, male participants completed a 2-week study protocol. The volunteers were assigned to one of the four groups that differed in luminance levels (27-285 cd/m^2^). Within the four groups, each volunteer was exposed to a low melanopic (LM) and a high melanopic (HM) condition. The two 17-h study protocols comprised 3.5 h of light exposure starting 4 h before habitual bedtime. Median pupil size was significantly smaller during HM than LM in all four light intensity groups. In addition, we observed a significant correlation between melanopic weighted corneal illuminance (melanopic equivalent daylight illuminance [mEDI]) and pupil size, such that higher mEDI values were associated with smaller pupil size. Using pupil size to estimate retinal irradiance showed a qualitatively similar goodness of fit as mEDI for predicting melatonin suppression. Based on our results here, it remains appropriate to use melanopic irradiance measured at eye level when comparing light-dependent effects on evening melatonin concentrations in healthy young people at rather low light levels.

The pupil is the aperture of the eye and regulates the amount of light reaching the retina. The pupil constricts in bright environments and dilates in dark ones (Crawford, 1936). However, as early as 1962, Bouma et al. could show that the pupil response is wavelength dependent (Bouma, 1962). Meanwhile, there is clear evidence that pupil size and pupil light reflex change not only with illuminance (Lucas et al., 2003) but also with spectral distribution (Bouma, 1962; Chung and Pease, 1999; Ostrin et al., 2017).

About 20 years ago, a new class of photoreceptors was discovered in the human retina. These photoreceptors are intrinsically sensitive to short-wavelength light at about 480 nm (intrinsically photosensitive retinal ganglion cells, ipRGCs) and, express the photopigment melanopsin (Bailes and Lucas, 2013; Berson et al., 2002; Dacey et al., 2005; Hattar et al., 2002; Provencio et al., 2000; Thapan et al., 2001). ipRGCs encode information for diverse brain areas, such as the olivary pretectal nucleus (OPN), to control pupil light responses (Lucas et al., 2001, 2003) and the internal master clock in the hypothalamus located in suprachiasmatic nuclei to mediate circadian photoentrainment (Benarroch, 2011; Daneault et al., 2016; Gaggioni et al., 2014; Hattar et al., 2003; LeGates et al., 2014). Previous research has shown that evening exposure to short-wavelength light (i.e., high melanopic irradiance) can not only constrict pupil size but also acutely suppress melatonin concentrations, reduce alertness levels and affect sleep architecture (e.g., Cajochen et al., 2005, 2011; Lockley et al., 2006; Münch et al., 2006; Santhi et al., 2012).

Since the spectral sensitivity curves of the photoreceptors broadly overlap and all types of photoreceptors contribute to non–image-forming (NIF) effects (Güler et al., 2008; Lucas et al., 2001; Schmidt et al., 2011) such as pupil control (for an overview, see (Spitschan, 2019)), Tsujimura et al. used a silent substitution technique that aims at selectively targeting one class of photoreceptors without affecting the other types of photoreceptors (Estévez and Spekreijse, 1982; Spitschan and Woelders, 2018; Tsujimura et al., 2010). Under photopic conditions, more than three primaries are required to match light conditions in brightness and color (i.e., L-, M-, S-cone excitation). A conventional LED screen, for example, does not allow to generate so-called metameric lighting conditions, since it only contains three primaries. Therefore, different types of devices have been specially developed, such as Maxwellian vision systems and Ganzfeld stimulators (for a review see, Barrionuevo et al., 2022), projectors (e.g., Allen et al., 2018; Spitschan et al., 2019), and multiprimary displays (Blume et al., 2022; Proß, 2019). The method of silent substitution has been used in the field of colorimetry for decades (Estévez and Spekreijse, 1982) and it provides mechanistic insights into selective photoreceptor contributions. This is possible because photoreceptor output is univariant, depending only on the amount of photons absorbed by the photopigments in each effectively color blind photoreceptor (Rushton, 1972; Spitschan and Woelders, 2018; Stockman and Sharpe, 2000). Therefore, lights with different spectra can lead to the same photoreceptor excitation.

Tsujimura et al. could show that melanopsin-stimulating signals contribute stronger to the pupillary pathway than L- and M-cone signals (Tsujimura et al., 2010). Recently, several studies have selectively modulated melanopic irradiance while keeping L-, M-, and S-cone-opic irradiance constant in the form of metameric lights to gain more mechanistic insights into the relevance of the melanopsin system in circadian physiology and sleep. All these studies consistently found that high melanopic light during the evening reduces salivary melatonin levels in comparison to low melanopic light (Allen et al., 2018; Blume et al., 2022; Schöllhorn et al., 2023; Souman et al., 2018). However, these studies calibrated their light settings at eye level and did not consider pupil size to estimate the amount of light reaching the retina, although smaller pupil sizes may result in less illumination of the retina and thus might reduce the non-visual effects of light. It has already been shown that pupil dilation may enhance relatively low light level (50-200 lx) induced melatonin suppression compared with freely constricting pupils (Gaddy et al., 1993). Moreover, Higuchi et al. (2008) found a positive correlation between percentage of melatonin suppression by light with pupil area during light exposure (1000 lx) (Higuchi et al., 2008).

Here, we aimed to compare the effects of evening light exposure, using light settings that differed maximally in melanopic irradiance (high melanopic [HM] vs low melanopic [LM]), on steady-state pupil sizes assessed over the course of the evening. Illuminance levels ranged from 7 to 89 lx, which is commonly experienced in the evening when watching TV (~10 lx), reading on a mobile phone (~20 lx), using a tablet (~40 lx), or working on a computer screen (~80 lx). Because we matched our light settings for the three cone types, we expected (1) smaller steady-state pupil sizes in HM compared with LM and (2) this in a dose-dependent manner as a function of melanopic equivalent daylight illuminance (mEDI). In addition, (3) we wanted to exploratively observe whether the combination of pupil area and melanopic radiance to estimate melanopsin-weighted retinal irradiance (i.e., simple product of pupil area and melanopsin-weighted light intensity emitted by the screen) is a better predictor of melatonin concentration than melanopic illuminance (i.e., mEDI) alone.

## Methods

### Participants

Data were recorded continuously between December 2019 and July 2021 with interruptions between mid-March and mid-May 2020 due to the global COVID-19 pandemic. A total of 72 healthy young men between the ages of 19 and 35 years (intensity 1: 24.5 ± 3.8 years; intensity 2: 25.4 ± 5.5 years; intensity 3: 24.3 ± 4.3 years; intensity 4: 24.7 ± 3.5 years) were included in the study. To avoid possible effects of the menstrual cycle on sleep (Driver et al., 1996) and melatonin secretion (Greendale et al., 2020), we did not include female participants (Vidafar and Spitschan, 2023). Participants were seen by a study physician and a graduated optometrist to exclude volunteers with visual impairments. Participants with monocular visual acuity <0.5 (Freiburg Visual Acuity Test Version 3.10.2 [Bach, 1996]), with color vision deficiencies (Ishihara < 17 of 21 plates [Ishihara, 1998], 100-hue error score >40, Farnsworth, 1943]), and with reduced stereoscopic vision (Lang II <200 arc sec [Lang and Lang, 1988]) were excluded from the study. Note that all inclusion and exclusion criteria of this study have been already published in Schöllhorn et al. (2023), where we reported melatonin, subjective alertness, and sleep latency data.

### Study Protocol

The study protocol was approved by the ethics commission northwest/central Switzerland (2019-00571) and conformed to the Declaration of Helsinki. All participants provided written informed consent and received compensation for participation. The study consisted of one habituation night and two experimental days. The experimental nights took place on the same day of the week and were usually exactly 1 week apart. Participants were balanced randomly and assigned to one of the four light intensity groups (*n* = 18, per group). Within each light intensity group, we counterbalanced the order of the two light conditions (HM and LM). The laboratory environment was controlled without any external time cues, including clocks, smartphones, and daylight. Note that the study protocol and light conditions have been already reported in Schöllhorn et al. (2023).

### Experimental Visits

**Figure 1.**
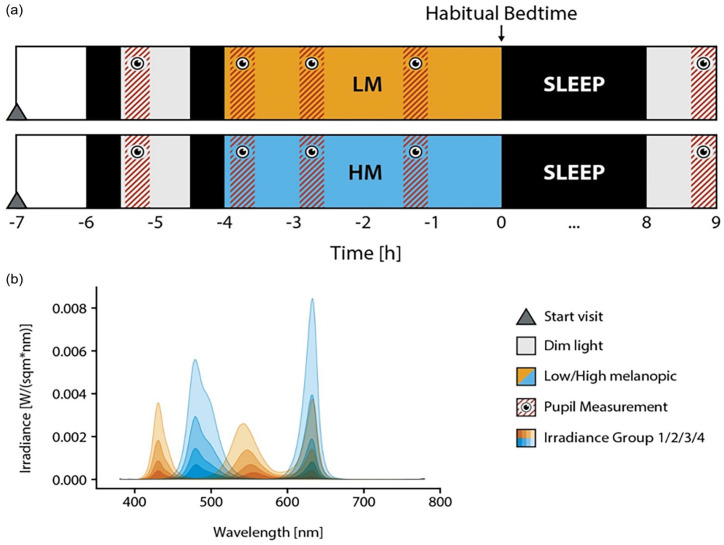
(a) Experimental protocol of the study. Each participant was exposed to low melanopic (LM) or high melanopic (HM) condition, respectively, for 3.5 h, starting 4 h before habitual bedtime. The light exposure was followed by an 8-h sleep and a 1-h dim light episode in the next morning. Before, during, and in the morning after light exposure, salivary melatonin was measured in half hourly intervals and cognitive performance (psychomotor vigilance task, go/no-go performance task, word-pair learning task) and objective alertness (Karolinska drowsiness test) were assessed, while pupil size was measured. The results of the cognitive tasks and objective alertness will be published elsewhere. (b) Spectra of the two experimental screen light conditions (W/[sqm*nm]). HM conditions are indicated by blue lines and LM by orange lines.

During the evenings of the two 17-h experimental visits, which only differed in the light condition (HM vs LM), participants came to the laboratory 7 h prior to habitual bedtime (see Figure 1). One hour of 67 lx fluorescent light (Philips Master TL5 HO 54W/830, CRI 80, 3000 K) was followed by two adaptation periods of complete darkness (~0.1 lx), separated by a period of dim light (~0-7 lx), which were used for photoreceptor sensitivity adaptation and baseline measurements. Participants were exposed to LM and HM conditions for approximately 3.5 h, starting 4 h before their usual bedtime. Before, during, and in the morning after LM and HM exposure, saliva samples were collected, electroencephalography was continuously recorded, participants performed cognitive tasks (i.e., psychomotor vigilance task, go/no-go performance task, word-pair learning task), subjective and objective alertness was assessed, and pupil size was measured. Here, we will only report on the results of the pupil data and the previously published melatonin data from Schöllhorn et al. (2023). During task-free periods, participants listened to prepared audio books and were instructed to look at the center of the screen. The light exposure was followed by 8 h of sleep in complete darkness and 1 h of dim light the next morning. Data on subjective sleep quality, which was assessed in the morning, will be published elsewhere.

### Light Conditions

The light was presented on a custom 27-inch visual display. The screen contained five different LED types (dominant wavelength: 430, 480, 500, 550, and 630 nm). The metameric screen was designed and calibrated using the method of silent substitution (Estévez and Spekreijse, 1982; Spitschan and Woelders, 2018) implemented in MATLAB (The Mathworks, Natick, MA). LEDs with wavelengths of 480, 500, and 630 nm were used for HM and LEDs with wavelengths of 430, 550, and 630 nm were used for LM. The light settings were matched in terms of cone excitation (L, M, and S) based on the 10° cone fundamentals using CIE S 026 (CIE S 026/E:2018, 2018). Adjustment of these five LEDs allowed for between 200% and 300% melanopsin contrast between the LM and HM conditions (for an overview of the light characteristics, see Table 1 and spectra are shown in Figure 1). Calibration measurements were performed with the JETI spectraval 1501 (JETI Technische Instrumente GmbH, Jena, Germany) at an eye level at a distance of 60 cm from the center of the screen. The spectrometer was inclined at an angle of 15° to the center of the monitor (Schöllhorn et al., 2023).

**Table 1.** Overview of luminances, irradiance-derived α-opic responses, α-opic equivalent daylight (D65) illuminances, mEDI ratios, and contrasts (Schöllhorn et al., 2023).

Condition	LM 1	HM 1	LM 2	HM 2	LM 3	HM 3	LM 4	HM 4
Luminance (cd/m^2^)	27.43	27.41	62.93	61.39	135.03	133	283.74	284.5
S-cone-opic irradiance (mW⋅m^−2^)	6.40	6.22	13.87	13.72	29.58	29.71	60.21	62.00
M-cone-opic irradiance (mW⋅m^−2^)	11.19	11.74	25.52	25.43	55.08	54.79	116.58	115.12
L-cone-opic irradiance (mW⋅m^−2^)	13.32	13.50	29.95	30.09	64.90	64.78	138.10	138.86
Rhodopic irradiance (mW⋅m^−2^)	6.87	18.70	16.47	40.39	38.09	86.81	87.00	180.89
Melanopic irradiance (mW⋅m^−2^)	4.91	20.11	11.70	43.51	27.46	93.27	64.17	193.62
mEDI (lx)	3.70	15.17	8.82	32.81	20.70	70.33	48.39	146.00
Ratio mEDI (HM/LM)	4.1		3.72		3.40		3.02	
Contrast mEDI (HM **−** LM)/LM (%)	310		272		240		202	

Abbreviations: mEDI = melanopic equivalent daylight illuminance; LM = low melanopic; HM = high melanopic. For the calculation of irradiance-derived α-opic responses and α-opic equivalent daylight (D65) illuminances, the *luox* app (https://luox.app/) was used (Spitschan et al., 2022).

### Pupil Size

The participants’ pupil diameter of the right eye was measured during the test sessions using an eye-tracking device (Pupil Labs GmbH, Berlin, Germany), which allows for the recording of pupil sizes at 200 Hz. When participants arrived in the laboratory, screen marker calibration choreography provided by Pupil Labs was performed. The algorithms of Pupil Core automatically run two detection pipelines in parallel, two-dimensional (2D) and three-dimensional (3D). The median pupil size of 20-min bins during each of the five test sessions (go/no-go task, Karolinska drowsiness test, and psychomotor vigilance task) was computed for statistical analyses. Therefore, we used the provided data of the 3D Pupil Detection (Dierkes et al., 2019; Swirski and Dodgson, 2013). Prior to statistical analysis, pupil measurements with a confidence of <0.6 and pupil diameters less than 2 mm and greater than 10 mm were excluded to remove artifacts and eye blinks. We then calculated the median pupil size per part for statistical analysis. Due to technical problems (i.e., loss of data due to storage problems), we lost the pupil recordings from three test visits (~2%) and a further seven test parts. In addition, we excluded data from 24 test parts (3.33%) due to poor data quality (i.e., <2% of available data). Therefore, for statistical analysis, the recordings of 674 (94%) test parts were used.

### Retinal Irradiance (Measured in Trolands)

Retinal irradiance cannot be measured directly in visual experiments. Therefore, to calculate a conventional retinal irradiance (measured in trolands), the product of luminance and pupil area is used (Thibos et al., 2018; Wyszecki and Stiles, 1982). Here, we additionally determined the α-opic retinal irradiances, measured in trolands, for the five human photoreceptor classes (melanopsin, rods, L-, M-, and S-cones) using spectral radiances, pupil areas, and spectral sensitivity functions based on the 10° cone fundamentals using CIE S 026 (CIE S 026/E:2018, 2018).

### Statistical Analyses

All statistical analyses were conducted in R (Version 4.1.1, R Core Team, 2020). Linear mixed model (LMM) analyses were performed separately for each light intensity group. “Light Condition” and “Time of day” were included as fixed effects and repeated measures per participant were modeled as random intercept. LMM analyses were followed by an analysis of variance (ANOVA) (Type = III) function. LMM analyses were calculated using the packages lme4 (Bates et al., 2015) and lmerTest (Kuznetsova et al., 2017). As an effect size measure, partial omega squared $\left(\omega_{p}^{2}\right)$ was calculated using the effectsize package (Ben-Shachar et al., 2020). It can be interpreted as follows: small effect: $\omega_{p}^{2}$ ≥ 0.01, medium effect: $\omega_{p}^{2}$ ≥ 0.06, and large effect: $\omega_{p}^{2}$ ≥ 0.14. A value of *p* < 0.05 was considered indicating statistical significance. Contrast tests for significant main effects of “Time of day” or significant interactions were performed using the emmeans package (Lenth, 2021) with Kenward-Roger degrees of freedom and a Tukey adjustment for multiple comparisons. Linear regression models were used to describe the relationship between mEDI and the observed variables.

## Results

### Steady-State Pupil Size

**Figure 2.**
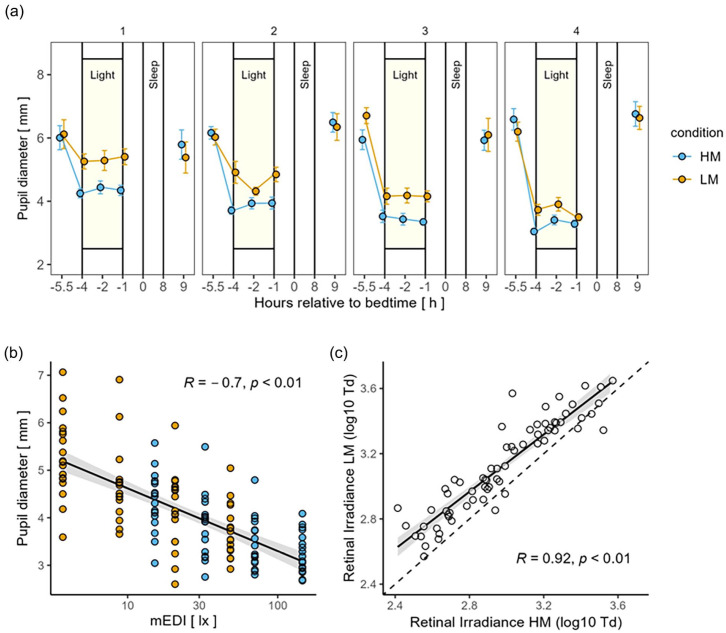
(a) Time course of pupil diameters during the low melanopic (LM: orange points and lines) and high melanopic (HM: blue points and lines) light conditions plotted against the hours relative to bedtime (h). Depicted are M ± 1 SEM. (b) Dose-response relationships with log10-transformed mEDI (lx) calculated for average pupil diameters during light exposure (LM: orange points, HM: blue points) and the corresponding regression line. (c) Correlation between log10-transformed retinal irradiance of LM and HM and the corresponding regression line. The gray bands represent the 95% confidence interval limits. Abbreviation: mEDI = melanopic equivalent daylight illuminance.

The time course of median pupil diameter is illustrated in Figure 2a. During light exposure, pupil diameter was significantly larger in the LM compared to the HM condition in all four light intensity groups (*p* < 0.001, large effects, see Table 2). The main factor “time of day” was only significant in light intensity group 4 (*F*_1,80_ = 3.21, *p* < 0.05, $\omega_{p}^{2}$ = 0.05). However, post hoc comparisons did not show significant differences (*p* > 0.05). Within the four light intensity groups, the approximately 3× reduction in melanopic irradiance caused an average increase in pupil size between 16% and 23%, depending on the different light intensity groups (i.e., intensity 1: 22.6%, intensity 2: 20.4%, intensity 3: 21.1%, and intensity 4: 15.6%). The interaction between “Light Condition” and “Time of day” was only significant in intensity group 2 (Light condition * time of day: F_2,81_ = 3.43, *p* = 0.04, $\omega_{p}^{2}$ = 0.19; *session 2: HM − LM: t*_(81)_ = −5.44, *p* < 0.001, *session 4: HM − LM: t*_(81)_ = −4.04, *p* = 0.002).

**Table 2.** Analysis of variance results and effect sizes $\left(\omega_{p}^{2}\right)$ .

Pupil Diameter	Light Condition (LM vs HM)	Time of day	Light Condition * Time of day
Intensity 1 (27 cd/m^2^)	***F*_1,82_** **=** **47.11, *p*** **<** **0.001**, $\omega_{p}^{2}$ = **0.84**	*F*_2,82_ = 0.24, *p* = 0.79	*F*_2,82_ = 0.17, *p* = 0.85
Intensity 2 (62 cd/m^2^)	***F*_1,82_** **=** **41.15, *p*** **<** **0.001**, $\omega_{p}^{2}$ **=** **0.67**	*F*_2,81_ = 1.94, *p* = 0.15	***F*_2,81_** **=** **3.43, *p*** **=** **0.04**, $\omega_{p}^{2}$ = **0.19**
Intensity 3 (134 cd/m^2^)	***F*_1,80_** **=** **36.79, *p*** **<** **0.001**, $\omega_{p}^{2}$ = **0.48**	*F*_1,79_ = 0.11, *p* = 0.90	*F*_1,79_ = 0.10, *p* = 0.91
Intensity 4 (284 cd/m^2^)	***F*_1,82_** **=** **21.97, *p*** **<** **0.001**, $\omega_{p}^{2}$ = **0.20**	***F*_1,80_** **=** **3.21, *p*** **<** **0.05**, $\omega_{p}^{2}$ = **0.05**	*F*_1,80_ = 1.82, *p* = 0.17

Abbreviations: LM = low melanopic; HM = high melanopic. Effect sizes can be interpreted as follows—small effect: $\omega_{p}^{2}$ ≥ 0.01, medium effect: $\omega_{p}^{2}$ ≥ 0.06, large effect: $\omega_{p}^{2}$ ≥ 0.14. A value of *p* < 0.05 was considered indicating statistical significance. Results with *p* < 0.05 are in bold.

Grouping the data for pupil size according to the mEDI resulted in a significant dose-response relationship (*R* = −0.7, *p* < 0.01), such that higher mEDI levels were associated with a smaller pupil size (see Figure 2b).

Retinal irradiances of HM versus LM showed a high correlation (*R* = 0.92, *p* < 0.01). Given that pupil size, and therefore retinal irradiance, was higher in LM than in HM within each light intensity group, the regression line was constantly shifted in a more positive direction (see Figure 2c).

### Alpha-Opic Weighted Retinal Irradiance Versus Alpha-Opic Weighted Corneal Irradiance

**Figure 3.**
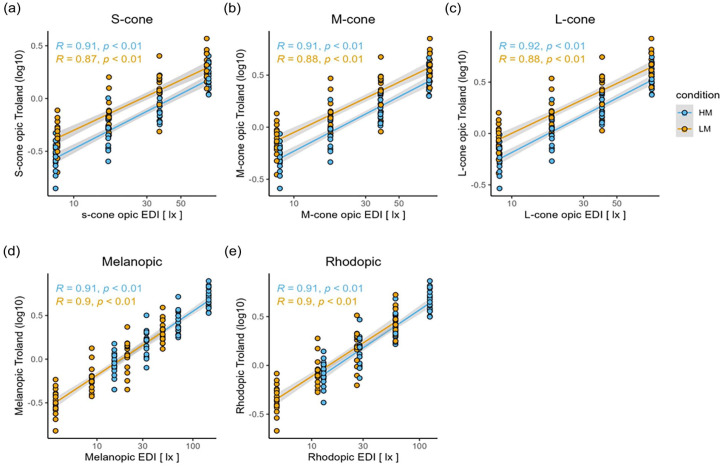
Correlation plots between α-opic equivalent daylight illuminances (EDIs) and log10-transformed α-opic trolands weighted for the different photoreceptor classes for the high melanopic (HM: blue) and low melanopic (LM: orange) conditions: (a) S-cones, (b) M-cones, (c) L-cones, (d) melanopsin, and (e) rhodopsin. The gray bands represent the 95% confidence limits.

Using α-opic spectra for weighting retinal (i.e., melanopic trolands) and corneal illuminance (i.e., mEDI) of the different photoreceptor types indicates higher melanopic trolands with increasing mEDI independent of the LM and HM condition (see Figure 3d and 3e). While our light conditions were matched for L-, M-, and S-cone-opic weighted EDIs, retinal irradiances for the three cone types were slightly higher during LM compared to HM, thanks to the difference in pupil size (see Figure 3a - 3c).

### Melanopsin-Weighted Retinal Irradiance as Predictor of NIF Effects

**Figure 4.**
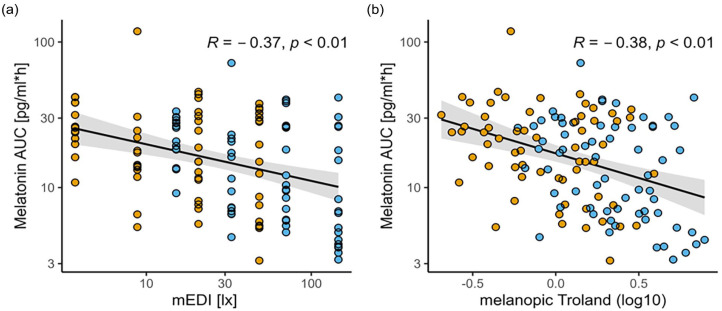
Dose-response relationships calculated for melatonin area under the curves (AUCs) during light exposure (pg/ml*h) (published in [Schöllhorn et al., 2023]) with log10-transformed (a) mEDI (lx) and (b) melanopic troland (Td). The gray bands represent the 95% confidence interval limits. Orange points depict individual values of the low melanopic and blue points show individual values of the high melanopic conditions. The gray bands represent the 95% confidence limits.

Although pharmacological pupil dilation has been shown to be a significant determinant of melatonin suppression, it is not a condition that occurs under natural lighting conditions (Giménez et al., 2022). Whether the use of melanopsin-weighted retinal irradiance by including pupil size is a better predictor of melatonin concentration than melanopic irradiance (i.e., mEDI) at relatively low light levels (<90 lx) remains to be determined. Therefore, we used the melatonin area under the curve (AUC) previously published by Schöllhorn et al. (2023) and compared linear fits using mEDI and melanopic trolands to predict evening melatonin AUCs. Dose-response relationships for melanopic EDI or melanopic trolands and melatonin AUCs showed similar fits by linear regression (*R* = −0.37, *p* < 0.01; *R* = −0.38, *p* < 0.01, respectively). Hence, the model fit could not be improved qualitatively by including pupil area (i.e., melanopic trolands) in the estimation of evening melatonin AUCs compared to mEDIs (see Figure 4).

## Discussion

Here, we aimed to quantify the specific impact of melanopic irradiance on steady-state pupil size in the evening during 4 h prior to habitual bedtime. The method of silent substitution using melanopsin-targeted stimuli to assess sustained pupil size (Viénot et al., 2010) and pupil response (e.g., Barrionuevo and Cao, 2016; Spitschan et al., 2014, 2019; Woelders et al., 2018; Zele et al., 2018) has been reported in several studies (see Supplementary Material for exploratory analysis of pupil size within the first 10 min of lights-on). However, to the best of our knowledge, previous studies assessing the evening profile of melatonin in humans while participants were exposed to melanopsin-directed stimuli (Allen et al., 2018; Blume et al., 2022; Souman et al., 2018) did not take into account the potential impact of pupil size differences between light conditions on NIF effects. In other words, they did not consider whether a smaller pupil size in high melanopic light compared to low melanopic light, resulting in less illumination of the retina, leads to a reduction in NIF effects. In principal, the capacity of higher melanopic stimuli to suppress melatonin could be offset by greater pupil constriction. Indeed, we found that pupil size was smaller in the HM condition than in the LM condition, in a dose-dependent manner as a function of melanopic EDI. This is consistent with previous studies suggesting that steady-state pupil size is mainly controlled by melanopsin (Gooley et al., 2012; Spitschan, 2019). Pupil constriction at higher melanopic EDI was calculated to reduce retinal irradiance, but the magnitude of this effect was small and it did not occlude the relationship between corneal melanopic irradiance and melatonin suppression.

Compared to two previous research studies, we did not find a robust time-dependent change in steady-state pupil size over the measurement period (Daguet et al., 2019; Van Egroo et al., 2019). However, both studies measured pupil size over a long period of time (i.e., 34 and 29 h) than in our study (i.e., 4 h) and in constant dim light under strictly controlled constant routine conditions. Thus, we assume that the time interval of pupil measurement in our study was too short to observe pupil size variations driven by the circadian processes and the sleep homeostasis. Besides light-induced and circadian phase–induced (Daguet et al., 2019; Van Egroo et al., 2019) changes in pupil size, steady-state pupil size can be affected by a plethora of factors such as sleepiness levels (Daguet et al., 2019; Danker-Hopfe et al., 2001; Morad et al., 2000; B. Wilhelm et al., 1998, 2001; H. Wilhelm et al., 1998; Yoss et al., 1970), fatigue (Morad et al., 2000), attentional effort (Massar et al., 2019), and cognition (Hess and Polt, 1960; Joshi and Gold, 2020; Kucewicz et al., 2018). As we measured pupil size during the assessment of cognitive tasks, this may have confounded our measurements.

We were interested in understanding whether the use of retinal versus corneal melanopic illuminance (melanopic trolands vs melanopic EDI) better predicts the attenuation of evening melatonin concentrations by light, which is the best proxy for quantifying light-dependent effects on circadian physiology in the evening and at night. Both models, melanopic trolands and melanopic EDI, showed a rather similar goodness of fit. According to our results here, it remains therefore appropriate to use melanopic irradiance measured at eye level when comparing light-dependent effects on evening melatonin concentration in healthy young people. However, it would be of great interest for future research on NIF effects to measure pupil size in situations where a reduced model prediction can be expected. This is likely to be the case in studies involving older participants, as pupil size decreases with age (Bitsios et al., 1996; Guillon et al., 2016; Telek, 2018) and older participants show reduced lens transmissibility particularly in the short-wavelength range (Artigas et al., 2012; Herbst et al., 2012; Kessel et al., 2010) both of which may lead to reduced NIF effects.

We cannot exclude the contribution of rods to pupil and melatonin responses, because of the rather low light levels in our study, especially in the light intensity group 1 (for detailed discussion, see [Schöllhorn et al., 2023]). In mice, rods have been shown to support circadian behavior across a wide range of light intensities (Altimus et al., 2010; Lall et al., 2010). Nevertheless, we refrained from silencing rods, as this would have drastically reduced melanopsin contrast (Spitschan and Woelders, 2018) and may have been of relatively low practical value, as both quantities covary in any realistic lighting scenario (Allen et al., 2018; Schöllhorn et al., 2023; Spitschan et al., 2021). To keep the nominal melanopsin contrast constant within the light intensity groups, spectral sensitivities were based on the “standard observer” CIE cone fundamentals (CIE S 026/E:2018, 2018) and not individually adjusted. Therefore, they may not have been metameric for all participants.

Watson and Yellott have developed a formula for estimating pupil size that takes into account the effects of luminance, the size of the adapting field, the age of the observer, and whether one or both eyes are adapted (Watson and Yellott, 2012). But, this model is based on *V*(λ) which only takes M- and L-cone excitation into account and ignores the contribution of S-cones and ipRGCs on steady-state pupil size (Spitschan, 2019). The results of our study underscore the importance of including spectral composition in the estimation of pupil size. However, the relatively small variation in pupil size within light conditions also suggests that pupil size in young participants is predictable when the melanopic irradiance of a light source is known. Rao et al. already developed a model that aims to integrate the contribution of ipRGCs on steady-state pupil size (Rao et al., 2017). Further research is needed to measure pupil size under realistic conditions, using light sources of different spectral composition, variable sizes, and in different age groups. In addition, as female volunteers were excluded, which clearly limits the generalizability of the present results, future research in females is needed.

In summary, our data identify melanopic irradiance as a valid parameter in young healthy participants for predicting pupil size at relatively low light levels (<90 lx). Here, the use of retinal compared to corneal melanopic illuminance showed qualitatively the same goodness of fit for predicting melatonin suppression. Hence, based on our results, it is still appropriate to use melanopic irradiance measured at eye level when comparing light-dependent effects on evening melatonin concentrations in healthy young people at the rather low light levels commonly experienced during evening screen use.

## Supplemental Material

sj-docx-1-jbr-10.1177_07487304241226466 – Supplemental material for The Impact of Pupil Constriction on the Relationship Between Melanopic EDI and Melatonin Suppression in Young Adult Males Supplemental material, sj-docx-1-jbr-10.1177_07487304241226466 for The Impact of Pupil Constriction on the Relationship Between Melanopic EDI and Melatonin Suppression in Young Adult Males by Isabel Schöllhorn, Oliver Stefani, Robert J. Lucas, Manuel Spitschan, Christian Epple and Christian Cajochen in Journal of Biological Rhythms
