# Supplementary material for: The Impact of Pupil Constriction on the Relationship Between Melanopic EDI and Melatonin Suppression in Young Adult Males
Source: J Biol Rhythms. 2024 Feb 13;39(3):282–94. doi: 10.1177/07487304241226466 (PMC11141089; doi:10.1177/07487304241226466)
Supplement: sj-docx-1-jbr-10.1177_07487304241226466 – Supplemental material for The Impact of Pupil Constriction on the Relationship Between Melanopic EDI and Melatonin Suppression in Young Adult Males [file sj-docx-1-jbr-10.1177_07487304241226466.docx]

# Supplementary Information

**The impact of pupil constriction on the relationship between melanopic EDI and melatonin suppression in young adult males**

Isabel Schöllhorn^1,2^, Oliver Stefani^1,3^, Robert J. Lucas^4^, Manuel Spitschan^5,6,7^, Christian Epple, Christian Cajochen^1,2*^

1 Centre for Chronobiology, Psychiatric Hospital of the University of Basel, Basel, Switzerland

2 Research Platform Molecular and Cognitive Neurosciences (MCN), University of Basel, Basel, Switzerland

3 Lucerne University of Applied Sciences and Arts, Horw, Switzerland

4 Centre for Biological Timing, School of Biology, Faculty of Biology Medicine and Health, University of Manchester, Manchester, UK

5 Translational Sensory & Circadian Neuroscience, Max Planck Institute for Biological Cybernetics, Tübingen, Germany

6 Chronobiology & Health, TUM Department of Sport and Health Sciences (TUM SG), Technical University of Munich, Munich, Germany

7 TUM Institute for Advanced Study (TUM-IAS), Technical University of Munich, Garching, Germany

*Corresponding author: Prof. Dr. Christian Cajochen, Centre for Chronobiology, Psychiatric Hospital of the University of Basel, Wilhelm Klein-Strasse 4002 Basel, Switzerland. Email: [christian.cajochen@upk.ch](mailto:christian.cajochen@upk.ch)

**Exploratory analysis: Pupil response after dark-adaptation**

Although it was not an initial aim of the study to measure the immediate pupil response after dark adaptation, we decided to include this exploratory analysis because it might help estimating the contribution of the other photoreceptor types during the initial phase after light onset. However, many participants had difficulty keeping their eyes open. Therefore, we often could not record sufficient data (i.e. >10%=20 measures per second) with good confidence > 0.6 (Intensity 1 HM: 66%, LM: 73%; Intensity 2 HM: 87%, LM: 84%; Intensity 3 HM: 78%, LM: 75%, Intensity 4 HM: 80%, LM: 81%).


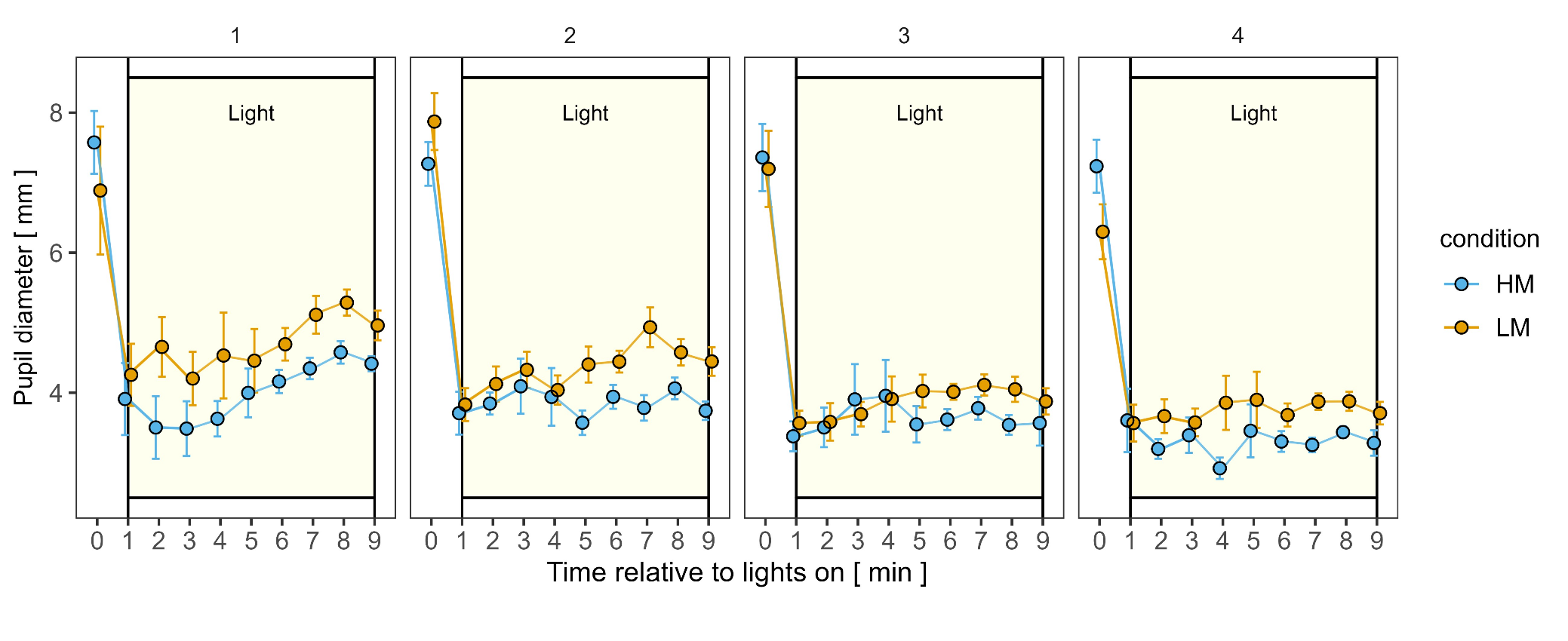


**Figure 1. A** Time course of pupil diameters during the low melanopic (LM: orange points and lines) and high melanopic (HM: blue points and lines) light conditions plotted against the time relative to lights on [h]. Depicted are means ±1SEM.

The post-illumination pupil response has previously been shown to be primarily driven by the melanopsin system. Gooley et al (2012) investigated the contribution of visual photoreceptors versus melanopsin by comparing sighted and blind (i.e. without rod and cone responses) participants. They found that after 10 minutes of exposure to polychromatic light there was no difference between the two groups, suggesting that pupil size after 10 minutes is mainly melanopsin dependent. However, they were also able to show that low irradiances (<13 log photons cm^-2^ s^-1^) can lead to a gradual increase in pupil size during brief light stimulation, which can last for at least 30 minutes. In contrast, exposure to higher irradiances (13.5 log photons cm^-2^ s^-1^) resulted in a very small change in pupil diameter over time. This is consistent with the results of our study, which showed only a redilatation of the pupil in the lowest light intensity group. Since Gooley and colleagues could only find this gradual increase in sighted subjects, this suggests that this increase is driven by rods and cones (Gooley et al., 2012). This suggests that when collecting pupil data to generate pupil models that include low light levels (i.e. ~<20 lx), exposure times of approximately 10 minutes after dark adaptation may be required to assess melanopsin-driven sustained pupil size.

Gooley, J. J., Ho Mien, I., St. Hilaire, M. A., Yeo, S.-C., Chua, E. C.-P., Van Reen, E., Hanley, C. J., Hull, J. T., Czeisler, C. A., & Lockley, S. W. (2012). Melanopsin and Rod–Cone Photoreceptors Play Different Roles in Mediating Pupillary Light Responses during Exposure to Continuous Light in Humans. *The Journal of Neuroscience*, *32*(41), 14242–14253. https://doi.org/10.1523/JNEUROSCI.1321-12.2012
